# BAF45D Downregulation in Spinal Cord Ependymal Cells Following Spinal Cord Injury in Adult Rats and Its Potential Role in the Development of Neuronal Lesions

**DOI:** 10.3389/fnins.2019.01151

**Published:** 2019-10-29

**Authors:** Zhenzhen Wang, Jian Huang, Chang Liu, Lihua Liu, Yuxian Shen, Cailiang Shen, Chao Liu

**Affiliations:** ^1^Department of Spine Surgery, The First Affiliated Hospital of Anhui Medical University, Hefei, China; ^2^School of Basic Medical Sciences, Anhui Medical University, Hefei, China; ^3^Department of Histology and Embryology, Anhui Medical University, Hefei, China; ^4^Institute of Stem Cell and Tissue Engineering, Anhui Medical University, Hefei, China; ^5^Institute of Clinical Pharmacology, Anhui Medical University, Hefei, China

**Keywords:** BAF45D, spinal cord injury, spinal cord ependymal cells, neurite, motor neuron

## Abstract

The endogenous spinal cord ependymal cells (SCECs), which form the central canal (CC), are critically involved in proliferation, differentiation and migration after spinal cord injury (SCI) and represents a repair cell source in treating SCI. Previously, we reported that BAF45D is expressed in the SCECs and the spinal cord neurons in adult mice and knockdown of BAF45D fail to induce expression of PAX6, a neurogenic fate determinant, during early neural differentiation of human embryonic stem cells. However, the effects of SCI on expression of BAF45D have not been reported. The aim of this study is to explore the expression and potential role of BAF45D in rat SCI model. In this study, adult rats were randomly divided into intact, sham, and SCI groups. We first explored expression of BAF45D in the SCECs in intact adult rats. We then explored SCI-induced loss of motor neurons and lesion of neurites in the anterior horns induced by the SCI. We also investigated whether the SCI-induced lesions in SCECs are accompanied by the motor neuron lesions. Finally, we examined the effect of BAF45D knockdown on cell growth in neuro2a cells. Our data showed that BAF45D is expressed in SCECs, neurons, and oligodendrocytes but not astrocytes in the spinal cords of intact adult rats. After SCI, the structure of CC was disrupted and the BAF45D-positive SCEC-derivatives were decreased. During the early stages of SCI, when shape of CC was affected but there was no disruption in circular structure of the SCECs, it was evident that there was a significant reduction in the number of neurites and motor neurons in the anterior horns compared with those of intact rats. In comparison, a complete loss of SCECs accompanied by further loss of motor neurons but not neurites was observed at the later stage. BAF45D knockdown was also found to inhibit cell growth in neuro2a cells. These results highlight the decreased expression of BAF45D in SCI-injured SCECs and the potential role of BAF45D downregulation in development of neuronal lesion after SCI in adult rats.

## Introduction

Traumatic SCI results in the death of neurons and glial cells, ultimately resulting in neurological deficits (Ahuja et al., 2017). One potential therapeutic strategy for repairing SCI involves a combination of neuroprotective and neuroregenerative treatments via the use of NSCs (Assinck et al., 2017).

The spinal cord ependymal cells (SCECs) that line the CC of all vertebrates are potential NSCs and play essential roles in the structure and physiology of the normal spinal cord (Jimenez et al., 2014; Moore, 2016). However, recent reports have cast doubt over whether the spinal cord ependymal region represents a neurogenic niche and suggest that this region would not be involved in cell replacement after the development of a lesion in adult humans (Alfaro-Cervello et al., 2014; Garcia-Ovejero et al., 2015; Paniagua-Torija et al., 2018). Furthermore, whether the transplantation of NSCs truly represents a viable clinical option, and can lead to the development of new therapeutic strategies for SCI, remains unclear (Assinck et al., 2017) and would require a deeper understanding of how the local environment influences neural repair (O’Shea et al., 2017) and whether we can develop novel combination strategies (Bunge, 2008; Becker et al., 2018). SCI results in damage to the neural network and causes loss of function. At present, there is no effective treatment for SCI. However, recent developments in stem cell research have fueled the development of regenerative therapy using NSC transplantation, thus making it possible to rebuild the destroyed neural circuits (Zhu et al., 2018).

Spinal cord ependymal cells have been shown to possess the capacity to differentiate into neurons and glia cells *in vitro* (Sabelstrom et al., 2014). After injury, the spinal cord environment appears to restrict the fate of SCECs to glial phenotypes. Evidence for this was reported in a previous study which found that most SCECs generated glial cells when grafted into the spinal cord, but formed neurons when placed into the hippocampus, a neurogenic niche (Shihabuddin et al., 2000). These glial phenotypes, which form the core of the glial scar (Cregg et al., 2014; Gregoire et al., 2015), are highly beneficial for recovery, as the glia scars may support the regeneration of axons and restrict both tissue damage and neural loss (Stenudd et al., 2015; Anderson et al., 2016). In a recent human clinical trial, human spinal cord NSC transplantation was shown to be safe and potentially efficacious in the treatment of chronic SCI (Curtis et al., 2018). Thus, a combination of stem cells and gene manipulation is highly likely to make a substantial contribution to the development of new therapies for SCI (Wang et al., 2019).

Several papers have reported that the promotion of neurite outgrowth provides an encouraging strategy for the potential treatment of SCI patients (Wu et al., 2016; Wang et al., 2017, 2018; Kucher et al., 2018). However, after SCI, the local microenvironment appears to govern the fate of the SCECs to mainly glial phenotypes, creating a challenge for the generation of new neurons (Becker et al., 2018). It has been reported that Noggin, a BMP antagonists expressed in SCECs, prohibits the SCECs from differentiating into glial cells and induces their differentiation into neurons (Lim et al., 2000). Consequently, researchers are currently trying to manipulate SCECs in an effort to facilitate neuronal differentiation (Duan et al., 2016). The neuron-specific class III beta-tubulin (beta-III-tubulin), a neuronal cytoskeleton protein, has been used to identify neurons and monitor neurite growth *in vitro* (Hu et al., 2015; Ahn and Cho, 2017). However, if the SCEC are related to *in vivo* neurite lesion and neuron loss after SCI in animal models has not been well-addressed. Previously, we identified that BAF45D protein, also known as DPF2, is expressed in the SCECs and neurons, but not astrocytes, of the spinal cords in adult mice (Liu et al., 2017). Research has shown that *Baf45d* mRNA is present in the developing cerebral cortex of mouse embryos on embryonic day 14 and that BAF45D protein is present in the hippocampus of adult mice (Gabig et al., 1998). BAF45D belongs to BAF45 family proteins, subunits of the BAF complex which includes BAF45A, BAF45B, BAF45C, and BAF45D (Lessard et al., 2007). In our previous work, we found that the knockdown of BAF45D resulted in a failure to induce the expression of PAX6, a neurogenic fate determinant (Ninkovic et al., 2013; Gotz et al., 2016), during the early neural differentiation of H9 cells induced by retinoid acid (Liu et al., 2017). Moreover, PAX6 is known to contribute to both embryonic and adult neurogenesis as a multifunctional regulator (Osumi et al., 2008). Since SCECs are defined as spinal cord NSCs and involved in the proliferation, differentiation, and migration of SCECs after SCI (Mothe and Tator, 2005), we thus want to explore expression of BAF45D during SCI. Furthermore, we wanted to ascertain whether the levels of BAF45D protein were affected by SCI, particularly in the SCECs, as this may imply that BAF45D plays a potential role in neuronal differentiation.

In this study, we investigated the expression of BAF45D in the SCECs of rats in intact, sham and SCI groups. Next, we evaluated the loss of motor neurons and the development of neurite lesion in the anterior horns following SCI. Moreover, we investigated whether the SCI-induced loss of SCECs are accompanied by the neuronal lesions. Finally, we examined the effect of *Baf45d* siRNA on the growth of neuro2a cells.

## Materials and Methods

### Animals and Ethics Statement

Female Sprague-Dawley (SD) rats (8 weeks of age, 220–250 g in body weight) were ordered from the Experimental Animal Centre of Anhui Province and used for breeding and surgical experiments. All animal experiments were approved by the Anhui Medical University Experimental Animal Ethics Committee. The acquisition and care of animals conformed to the NIH Guide for the Care and Use of Laboratory Animals (National Institutes of Health Publications, No. 80-23, revised 1978).

### The Establishment of SCI Model

The SCI model was established according to a previous protocol (Chen et al., 2017). SD rats were randomly divided into three groups: (i) an intact group (received no treatment), (ii) a sham group (received laminectomy without SCI), and (iii) a SCI group (received SCI for 10 and 14 days, respectively). For surgical treatment, the animals were immobilized on a surgical panel in a prostrate position. After disinfection, we used sterile drapes to cover the incision site. We then performed a midline spinal incision from T9 to T11 and stripped away the paraspinal muscles. After the spinal cord had been exposed by laminectomy, we placed the animals into a traumatic SCI device. The device measured the movement of a 10-g rod dropped vertically from a 25 mm height onto the spinal cord at T10. Local edema, and even hematoma, was immediately evident at the impact site on the spinal cord. Lower limb spasm, and tail flicking indicated the successful establishment of the SCI model. Following surgery, the incision was irrigated with normal saline and the spinal cord was recovered with subcutaneous fascia. Then, the incision was closed layer-by-layer. The rats were then housed in an incubation hatch until they regained consciousness; temperature was controlled at 22–25°C and the hatch was well-ventilated. Rats were provided with water and food *ad libitum.* Artificial bladder urination was performed three times a day until micturition was restored.

### Hind Limb Motor Function Assessment

Rats were evaluated using the BBB open-field-gait assessment, a 21-point scale system developed to assess hind limb locomotor function in rats following SCI. Statistical analysis of the BBB scores involved 5 rats in the intact group, 5 rats in the sham group, and 10 rats in the SCI group. In addition, of the 10 rats in the SCI group, 5 rats were scored on day 10 and 5 rats were scored on day 14. The score for intact (pre-injury) hind limb motor function was defined as 21 while the score for the complete loss of motor function was defined as 0. Because we wanted to study both acute and subacute scoring following SCI, we evaluated spinal cord function on days 1, 3, 7, 10, and 14 after SCI, as described in a previous report (Lang et al., 2015).

### Tissue Preparation

Rats from each group were anesthetized with 10% chloralhydrate and sacrificed on days 10 and 14 after SCI. For morphological assessment, the rats were sequentially perfused with normal saline and then fixed with 4% paraformaldehyde (PFA). For biochemical assays, the tissues were isolated and stored at −80°C to await further preparation, as previously described (Lacroix et al., 2014). In brief, the tissues were first dissected and then post-fixed overnight in 4% paraformaldehyde at 4°C. Serial sections of 5 μm thickness were cut using a Leica microtome and mounted on CITOGLAS adhesion microscope slides.

### Cell Culture and siRNA Transfection

Neuro2a cells were cultured as previously described (Liu et al., 2007) and siRNA transfection was performed according to our previous protocol (Liu et al., 2015). In brief, neuro2a cells were cultured overnight in Dulbecco’s modified Eagle’s medium (DMEM) (Hyclone) containing 10% newborn calf serum (BI). Cells were then washed with Opti-MEM (Invitrogen) and then transiently transfected with negative control (NC) siRNA and Baf45d siRNA using Lipofectamine@ 2000 reagent (Invitrogen) in Opti-MEM without serum. The same volume of DMEM, containing 10% newborn calf serum, was added to the culture media 6 h after transfection. We then evaluated growth of the neuro2a cells by counting the number of cells present at 24, 48, and 72 h. Cells were harvested for immunoblotting 72 h after the transfection.

### Immunohistochemical (IH) Assay

Immunohistochemical (IH) assay was performed as described previously (Lacroix et al., 2014). Tissue sections were first blocked with 3% goat serum and then incubated with rabbit anti-BAF45D (1:100, Proteintech, Chicago, IL, United States), rabbit-anti-NESTIN (1:100, Sigma, St. Louis, MO, United States), mouse anti-NEUN (1:100, Millipore, Belecula, CA, United States) and mouse anti-beta-III-tubulin (1:200, Arigo) antibodies overnight at 4°C. Positive immunostaining was then detected using a NIKON Eclipse 80i fluorescent microscope and a NIKON Eclipse Ti-S inverted fluorescence microscope. Specific details of the animals and tissues used for IH are given in Supplementary Table S1.

### Immunofluorescence (IF) Assay

Immunofluorescence (IF) assay was performed according to our previous protocol (Liu et al., 2017). The samples were first incubated with rabbit anti-BAF45D (1:100, Proteintech, Chicago, IL, United States) together with mouse anti-NEUN (1:100, Millipore, Belecula, CA, United States), mouse anti-MBP (1:500, Abcam) and mouse anti-GFAP (1:100, Proteintech, Chicago, IL, United States) overnight at 4°C. After washing in PBS, the samples were then incubated with Alexa Flour-488 anti-mouse (1:500) and Alexa Fluor-594 anti-rabbit (1:500) secondary antibodies. Nuclei were counterstained with DAPI. The IF assay for cultured cells was performed in accordance with our previous report (Liu et al., 2007) and positive staining was visualized with a NIKON Eclipse 80i fluorescence microscope and a NIKON Eclipse Ti-S inverted fluorescence microscope.

### Immunoblotting (IB) Assay

Lysates were prepared from fresh tissues and cells and then subjected to immunoblotting (IB) in accordance with a previously described protocol (Liu et al., 2017). Proteins were then detected using a range of antibodies: mouse anti-GFAP (1:2000, Proteintech, Chicago, IL, United States), mouse anti-NEUN (1:500, Millipore, Belecula, CA, United States), rabbit anti-BAF45D (1:500, Proteintech, Chicago, IL, United States), mouse anti-beta-III-tubulin (1:1000, Arigo) and rabbit anti-GAPDH (1:2000, Proteintech, Chicago, IL, United States). Specific details of the antibodies used for IF and IB are given in Supplementary Table S2.

### Statistical Analysis

Image J software was used to analyze histological data and determine the number of immune-positive cells and neurites. Statistical analysis involved one-way analysis of variance (ANOVA) and was carried out using SPSS 17.0 software, unless stated otherwise. Proportional data were analyzed by the Mann–Whitney *U*-test using SPSS 17.0 software.

## Results

### BAF45D Is Expressed in the SCECs and Neurons in Intact Adult Rat Spinal Cords

**FIGURE 1:**
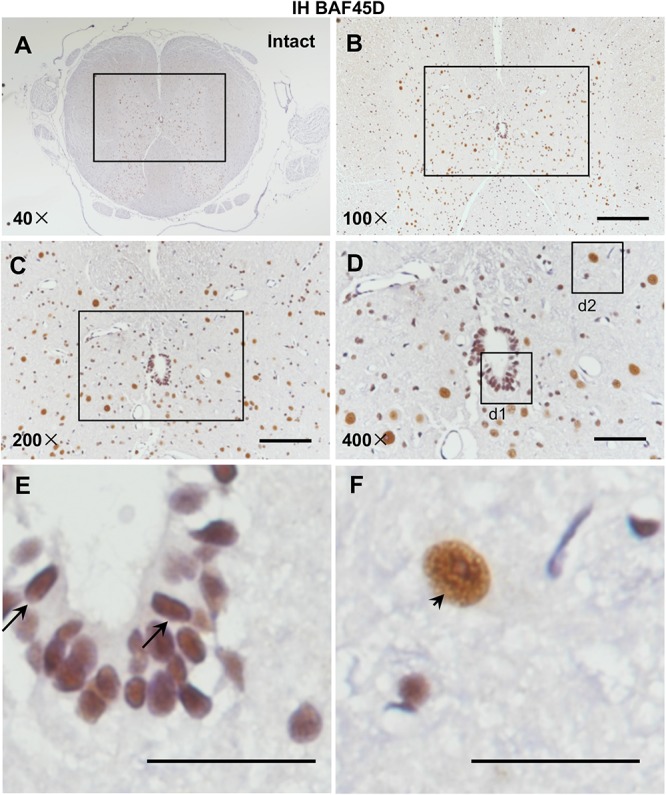
Expression of BAF45D in the SCECs and neurons of intact spinal cords from adult rats. **(A–E)** Immunohistochemical assays were performed using anti-BAF45D antibodies on histological sections prepared from the intact spinal cords of adult rats. **(B)** Shows a 100× magnification of the inset shown in **(A)**, while **(C)** shows a 200× magnification of the inset shown in **(B)**. **(D)** Represents a 400× magnification of the inset shown in **(C)** while **(E,F)** represent 2600× magnification of insets d1 and d2, respectively. Scale bars: 200 μm **(B)**, 100 μm **(C)**, 50 μm **(D)**, and 25 μm **(E,F)**.

Currently, rat SCI model has been used by researchers throughout the world to study repair strategies of SCI (Kim et al., 2017). However, no data address expression of BAF45D in rat spinal cord. Because we want to establish a rat SCI model and investigate structural and functional role of BAF45D in the model, we therefore decided to first examine expression of BAF45D in adult rat SCECs and neurons. We performed IH assay using the cross sections of intact spinal cords from adult rats (Figures 1A–F). The results indicated that BAF45D is expressed in almost all the SCECs (Figure 1E, arrows) and the neurons (Figure 1F, arrowhead).

**FIGURE 2:**
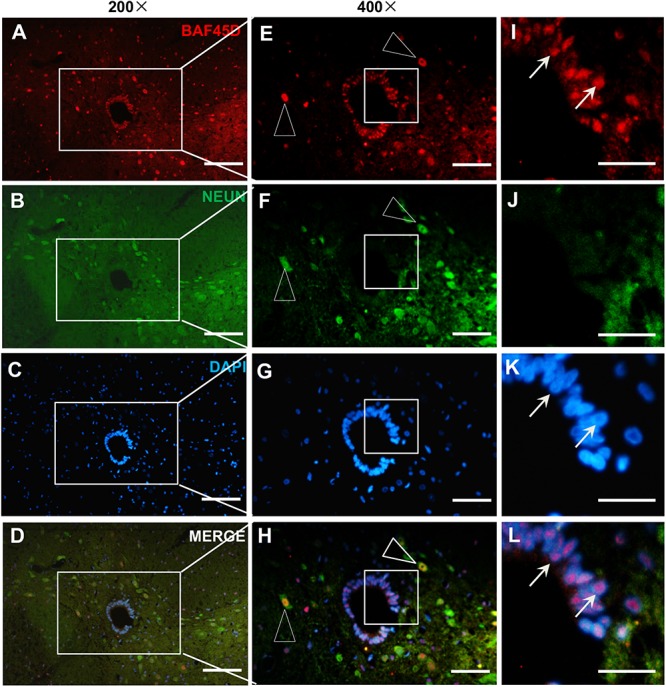
Expression of BAF45D and NEUN in the SCECs and neurons of intact spinal cords from adult rats. **(A–D)** Immunofluorescence assays were performed on histological sections prepared from the intact spinal cords of adult rats using anti-BAF45D and anti-NEUN antibodies. **(E–H)** Show 400× magnification of the insets shown in **(A–D)**, respectively. The triangles indicate neurons positive for both BAF45D and NEUN. **(I–L)** represent 1200× magnification of the insets shown in **(E–H)**, respectively. The arrows indicate SCECs positive for BAF45D. Scale bars: 100 μm **(A–D)**, 50 μm **(E–H)**, and 25 μm **(I–L)**.

To further address if BAF45D is expressed in the spinal cord neurons, we performed IF assay using anti-BAF45D and anti-NEUN antibodies (Figures 2A–L) and found that coexpression of BAF45D and NEUN, a neuron marker protein, can be identified in the neurons (Figures 2E,F,H, triangles). The SCECs are positive for BAF45D but not for NEUN (Figures 2I,J,L, arrows).

These results suggest that BAF45D is expressed in the SCECs and neurons in intact adult rat spinal cords.

### Spinal Cord Oligodendrocytes Express BAF45D While Astrocytes Express Little or No BAF45D

**FIGURE 3:**
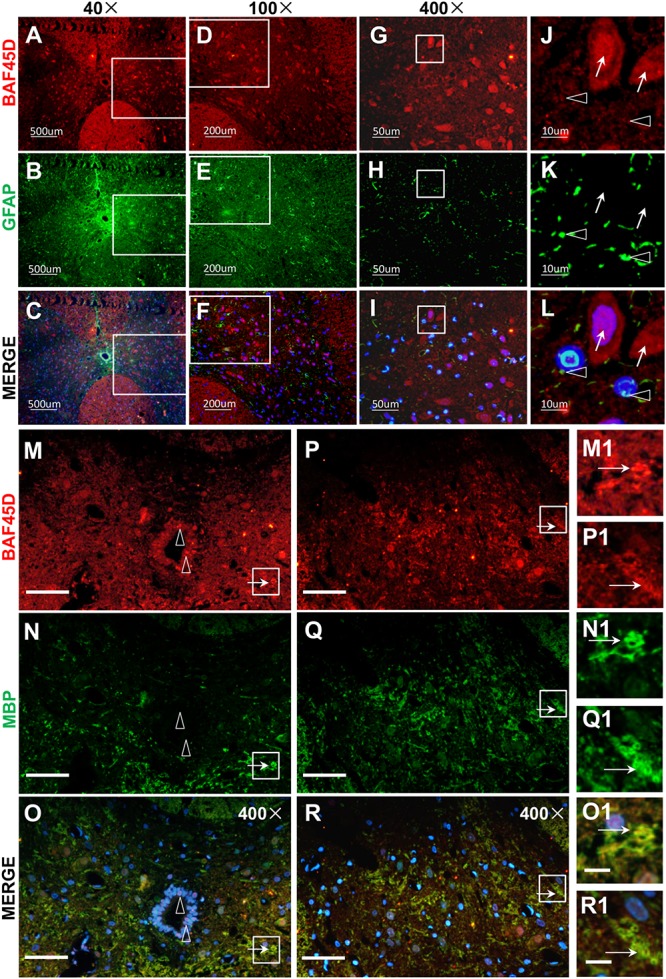
BAF45D was expressed in oligodendrocytes but not astrocytes in intact adult rat spinal cords. **(A–L)** Immunofluorescence assays were performed on histological sections prepared from intact adult rat spinal cords using anti-BAF45D and anti-GFAP antibodies **(A–C)**. **(D–F)** Represent 100× magnification of the insets shown in **(A–C)**, respectively. **(G–I)** Represent 400× magnification of the insets shown in **(D–F)**. **(J–L)** Represent 2000× magnification of the insets shown in **(G–I)**. BAF45D immune-positive cells are indicated by arrows while GFAP immune-positive cells are indicated by triangles. Scale bars: 500 μm **(A–C)**, 200 μm **(D–F)**, 50 μm **(G–I)**, and 10 μm **(J–L)**. **(M–R)** Immunofluorescence assays, using anti-BAF45D and anti-MBP antibodies, were performed on histological sections showing CC areas **(M–O)** and non-CC areas **(P–R)** in intact spinal cords from adult rats. **(M1–O1)** Represent 1200× magnification of the insets shown in **(M–O)**. **(P1–R1)** Represent 1200× magnification of the insets shown in **(P–R)**. Triangles indicate BAF45D-positive SCECs **(M–O)** while arrows indicate the colocalization of BAF45D with MBP **(M1–R1)**. Scale bars: 50 μm **(M–R)** and 10 μm **(O1,R1)**.

Next, to check the expression of BAF45D in spinal cord glial cells, we performed IF assay using anti-BAF45D and anti-GFAP antibodies (Figures 3A–L). The results revealed that BAF45D is expressed in some of the cells (Figures 3A,D,G,J, red), which were not positive for the GFAP, an astrocyte marker protein (Figures 3J–L, arrows). GFAP was detected in the astrocytes in cross sections (Figures 3B,E,H,K, green). Furthermore, little or no expression of BAF45D was found in the cells that were positive for GFAP (Figures 3K,L, triangles). Besides, we performed IF assay using anti-BAF45D and anti-MBP antibodies (Figures 3M–R). The results indicate that colocalization of BAF45D (Figures 3M,O,P,R,M1,O1,P1,R1, red) with MBP (Figures 3N,O,Q,R,N1,O1,Q1,R1, green), an oligodendrocyte marker located on myelin sheath membrane (Ganz et al., 2017), was detected in the rat spinal cords.

These results suggest that BAF45D is expressed in spinal cord oligodendrocytes but not astrocytes.

### BAF45D Is Downregulated in the Spinal Cord Lesion Site After SCI in Adult Rats

**FIGURE 4:**
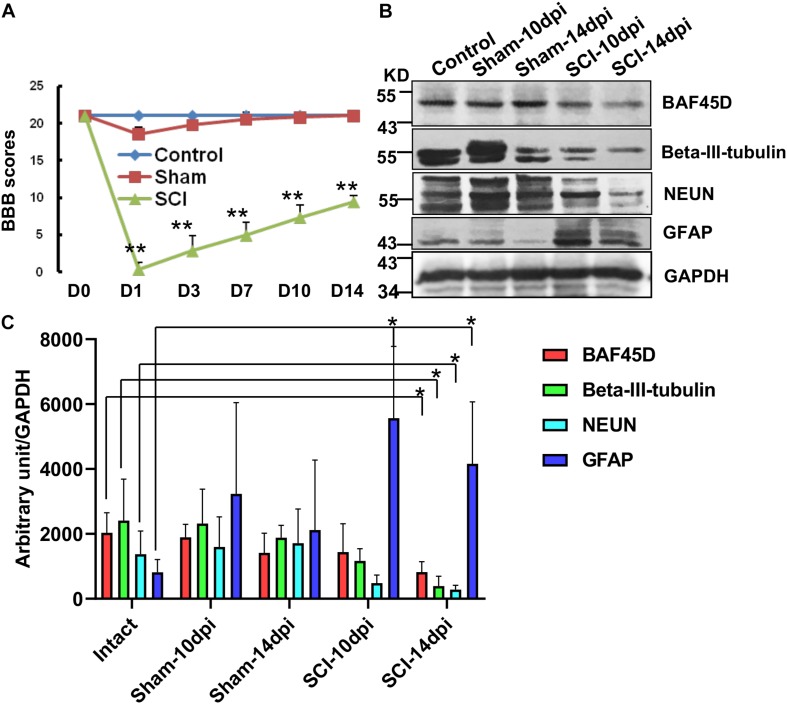
Downregulation of BAF45D at lesion sites following SCI in adult rats. **(A)** Hindlimb movements were evaluated using the 21-point BBB rating scale. **(B)** The expression of neural proteins was examined by IB assays using the indicated antibodies in intact and injured spinal cord tissues. **(C)** IB assays were replicated four times, and protein levels were quantified by Image J software. Protein levels (expressed in arbitrary units) were normalized to the levels of GAPDH and were subjected to independent *t*-tests using SPSS 17.0 software. ^∗^*P* < 0.05, as compared with the intact group.

To further address the role of BAF45D in structural function of spinal cord disease, we established a SCI model and explored expression of BAF45D in the injured T10 segment after SCI in adult rats. Hindlimb movements were evaluated using a 21-point BBB rating scale. The scores of SCI model rats were significantly different from that of normal and sham rats (Figure 4A). The IB assay results indicate BAF45D was decreased by SCI lesion. While a glial marker protein, GFAP, was increased by the SCI lesion, two neuron marker proteins, NEUN and beta-III-tubulin, were both decreased after SCI (Figure 4B). Statistical analysis further showed significant downregulation of BAF45D, NEUN and beta-III-tubulin 14 days after SCI (Figure 4C). Through IH assay, we found the lesion sites are composed of many non-neuronal cells (Supplementary Figure S2). The weak expression of BAF45D in non-neuronal cells was also observed (Supplementary Figure S3).

These data suggest that the downregulation of BAF45D is accompanied by neuronal loss after SCI in adult rats.

### BAF45D Is Downregulated in SCECs After SCI in Adult Rats

**FIGURE 5:**
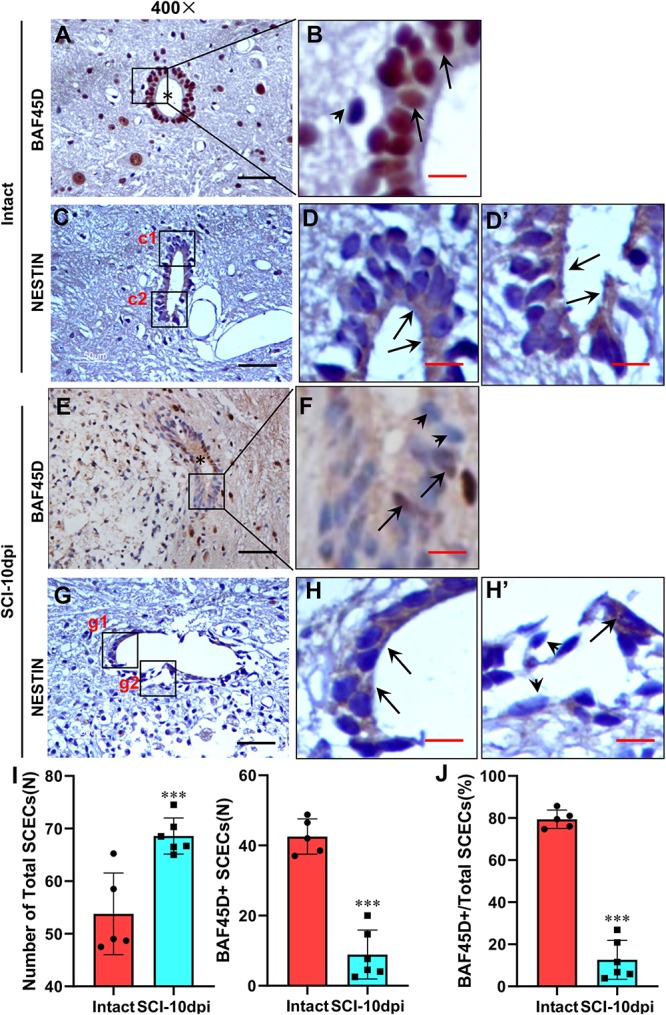
Downregulation of BAF45D in SCECs following SCI in adult rats. **(A,B)** Expression of BAF45D in the SCECs of intact spinal cord. **(B)** Represents a 2000× magnification of the inset shown in **(A)**. Asterisks indicate CC **(A)** while arrows indicate BAF45D-positive nuclei in SCECs. **(C–D’)** Expression of NESTIN in the SCECs of intact spinal cord. **(D,D’)** Represent 2000× magnification of insets c1 and c2 shown in **(C)**. Arrows indicate NESTIN-positive cytoplasm. **(E,F)** Expression of BAF45D in the SCECs of injured spinal cord. **(F)** Represents a 2000× magnification of the inset shown in **(E)**. The asterisk indicates injured CC **(E)** while arrows indicate BAF45D-positive nuclei; the arrowheads indicate BAF45D-negative nuclei in ependymal cells **(F)**. **(G–H’)** Expression of NESTIN in the SCECs of injured spinal cord. **(H,H’)** Represent higher magnifications of insets g1 and g2 shown in **(G)**. The arrows indicate NESTIN-positive cytoplasm. The arrowheads indicate little or no expression of NESTIN. Scale bars: 25 μm **(A,C,E,G)** and 5 μm **(B,D,D’,G,H,H’)**. **(I,J)** Tissue sections from five rats (*n* = 5) in the intact group and six rats (*n* = 6) in the SCI-10dpi (days post-injury) group were quantified using Image J software. Statistical analysis of the total numbers of SCECs and the proportion of BAF45D-positive cells in SCECs before and after SCI are shown **(I)**. Data represent mean ± SD. Statistical analysis involved one-way ANOVA and was carried out using SPSS 17.0 software. The proportion (%) of BAF45D-positive cells among the total number of SCECs in intact and injured spinal cords are shown **(J)**. Data represent mean ± SD. Statistical analysis involved the Mann–Whitney *U*-test and was carried out with SPSS 17.0 software. ^∗∗∗^*P* < 0.01, as compared with controls.

Next, we aimed to ascertain whetherBAF45D is downregulated in SCECs after SCI. Immunohistochemical assays demonstrated the expression of the BAF45D in the SCECs within the CC of the intact spinal cord (Figures 5A,B). We also checked expression of BAF45D in SCECs within SCI-injured CC (Supplementary Figures S1, S2). As shown in Figure 5E and Supplementary Figure S2G, the SCI-induced lesion site is located near to the CC. As a result, although CC was preserved in the SCI-injured spinal cord (Figures 5E,F), the shape of the CC (Figure 5E) was different from that of the intact spinal cord (Figure 5A). In the intact CC, almost all the ependymal cells are BAF45D-positive (Figure 5B, arrows). However, it was clear that SCI induced the downregulation of BAF45D in some SCECs (Figure 5F, arrowheads), while only a small number of BAF45D-positive SCECs were still detected (Figure 5F, arrows). NESTIN has been used as a marker of SCECs (Cawsey et al., 2015; Moore, 2016). The SCECs in both intact and injured spinal cords were also identified by IH assay using anti-NESTIN antibodies (Figures 5C–D’,G–H’). However, NESTIN expression in some of the SCI-affected SCECs (Figures 5H,H’, arrows) seems decreased as compared to that of the intact SCECs (Figures 5D,D’, arrowheads). Moreover, by statistical analysis, SCI induced a significant increase of total SCECs and a significant decrease of the BAF45D-positive SCECs (Figures 5I,J).

These results suggest that BAF45D expression is downregulated in some of the SCECs within CC despite the proliferation response of SCECs induced by SCI.

### SCI Exerted Deleterious Effects on SCECs and the Structure of the CC, Induced Neurite Lesions, and Led to a Loss of Motor Neurons

**FIGURE 6:**
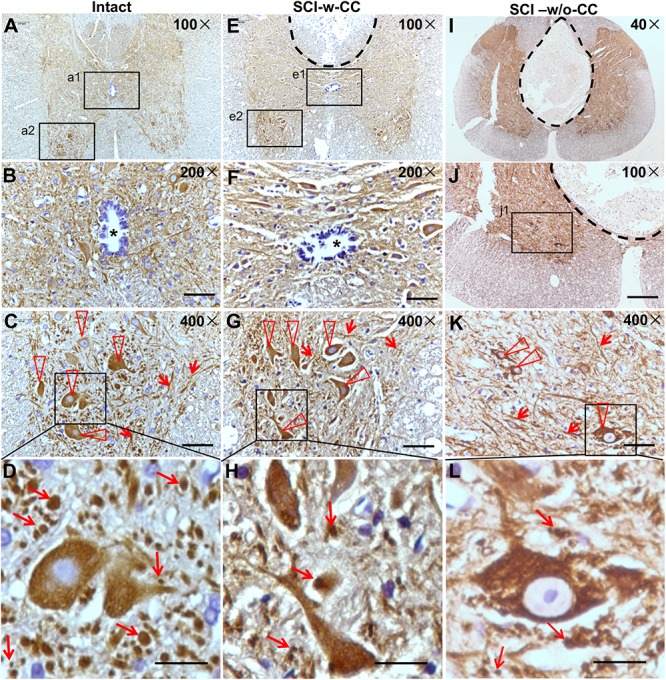
The organization of SCECs were related to neuronal lesions in injured spinal cords. **(A–D)** Expression of beta-III-tubulin in intact spinal cord neurites. **(B,C)** Represent 200× and 400× magnifications of insets a1 and a2 shown in **(A)**, respectively. The asterisk in **(B)** indicates an intact CC. **(D)** Represents a ∼1730× magnification of the inset shown in **(C)**. Beta-III-tubulin-positive motor neurons and neurites are indicated by triangles and arrowheads **(C)**. Beta-III-tubulin-positive neurites (CS-neurites) are indicated by arrows **(D)**. **(E–H)** Expression of beta-III-tubulin in injured spinal cord neurites. The region with a dashed line indicates the lesion site. **(F,G)** Represent 200× and 400× magnifications of insets e1 and e2 shown in **(E)**, respectively. The asterisk in **(F)** indicates a CC that is not damaged. **(H)** Represents a 1700× magnification of the inlet shown in **(G)**. Triangles indicate motor neurons **(G)** while arrows indicate CS-neurites **(H)**. **(I–L)** Expression of beta-III-tubulin in the neurites of neurons in injured spinal cords in which the CCs were damaged. **(L)** Represents a higher magnification of the inset shown in **(K)**. Scale bars: 50 μm **(B,F)**, 100 μm **(J)**, 25 μm **(C,G,K)**, and 10 μm **(D,H,L)**. Triangles indicate motor neurons **(K)** and arrows indicate CS-neurites **(K,L)**.

Beta-III-tubulin is a marker protein for studying neurite outgrowth in cultured neurons (Zhou et al., 2014). To characterize neurite out growth in the spinal cords *in vivo*, we first performed IHC assay using cross sections of intact and SCI-injured spinal cords (Figure 6 and Supplementary Figures S3, S4). The beta-III-tubulin positive signals were detected in the gray matter (Figure 6A). An oval CC is shown in the center area (Figure 6B, asterisk). In the anterior horn, while beta-III-tubulin is expressed in both cell bodies (Figure 6C, triangles) and threadlike neurites (Figure 6C, arrowheads), many intercellular granule-like structures are immunopositive for beta-III-tubulin (Figure 6D, arrows). We speculated that these granule-like structures may represent cross sections of the neurites. Therefore, we named the granule-like structures as CS-neurites. We next performed IH assay using cross sections of the injured spinal cords and found clearly the lesion sites, which are shown by a dashed line (Figure 6E). Even the lesion site does not reach to the CC, the CC was preserved (SCI-w-CC) and the shape of it became flattened (Figure 6F, asterisk). To our surprise, although the expression of beta-III-tubulin was still detected in cell bodies and thread-like neurites (Figure 6G, triangles and arrowheads), the number of the CS-neurites were decreased (Figure 6H, arrows). Moreover, the number of neurons in the anterior horn of injured spinal cord (Figure 6E) was also decreased as compared to that of intact spinal cords (Figure 6B).

We next asked if the preserved structure of CC is accompanied by neuronal lesion. We thus performed IH assay using cross sections of injured (Figures 6I–L) spinal cords, of which the CC disappeared (SCI-w/o-CC) and a well-developed non-neural lesion core formed (Figure 6I and Supplementary Figure S5). In the anterior horn (Figure 6J), the beta-III-tubulin immunopositive cell bodies (Figure 6K, triangles) and thread-like neurites (Figure 6K, arrowheads) were identified. We found that both the CS-neurites and the motor neurons of injured spinal cords without a CC were decreased significantly as compared to those of intact spinal cords. Moreover, although disappearance of CC has no significant effect on the number of the CS-neurites in injured spinal cord, the loss of the motor neurons in spinal cords in the absence of CC (Figures 6K,L) is more significant than that in spinal cords with preserved CC structure.

**FIGURE 7:**
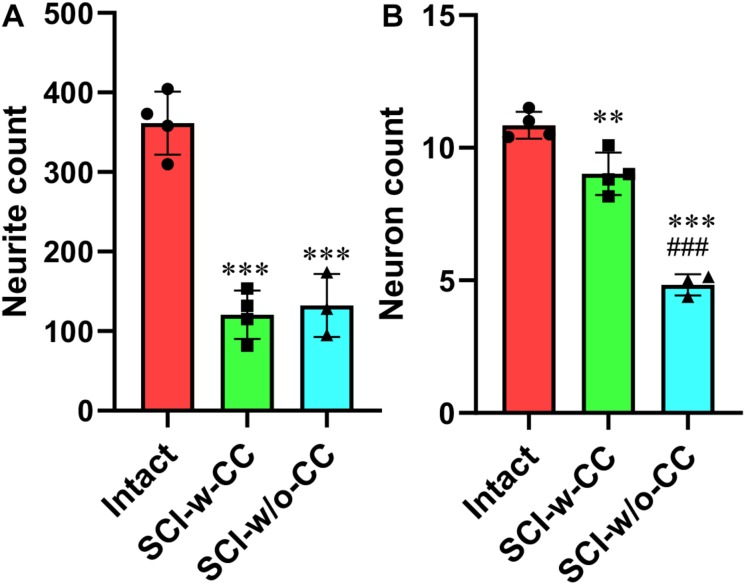
Statistic data relating to the reduction in beta-III-tubulin-positive CS-neurites and motor neurons. Beta-III-tubulin positive neurites **(A)** and motor neurons **(B)** were quantified using Image J software. Statistical analysis was then performed to compare the intact, SCI-w-CC and SCI-w/o-CC groups. Data represent mean ± SD. Statistical analysis involved one way ANOVA and was carried out with SPSS 17.0 software. ^∗∗^*P* < 0.01, ^∗∗∗^*P* < 0.001, as compared with the intact group. ###*P* < 0.001, as compared with the SCI-w-CC group.

Statistical analysis showed that while SCI induced a significant decrease in CS-neurites (Figure 7A) and motor neurons (Figure 7B), the disappearance of CC structure induced further damage in the motor neurons but not the CS-neurites, thus suggesting that SCECs, and in particular the organization of the SCECs, may play essential roles in the formation of neuronal lesion in the spinal cord after SCI.

### Knockdown of BAF45D Inhibits Cell Growth in Neuro2a Cells

**FIGURE 8:**
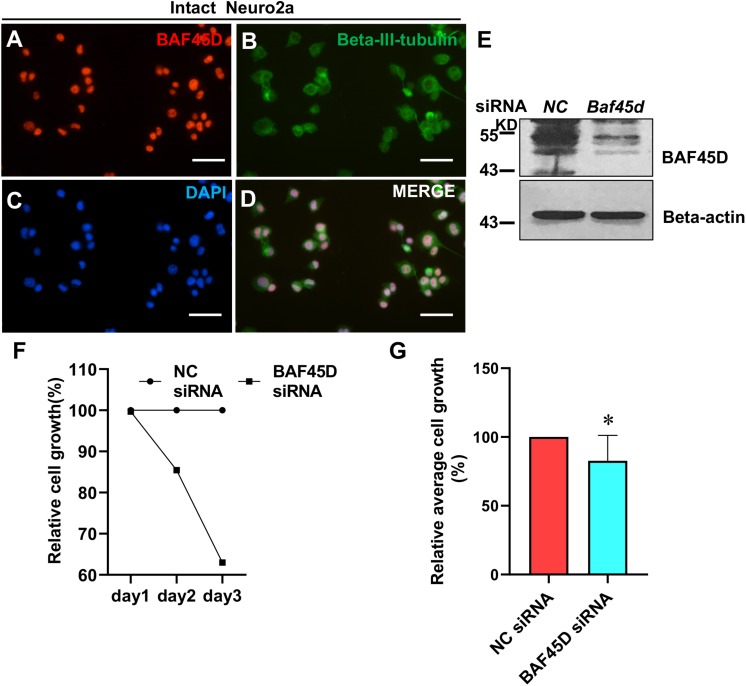
The knockdown of BAF45D inhibited cell growth in neuro2a cells. **(A–D)** Neuro2a cells were subjected to IF assays using anti-BAF45D and anti-beta-III-tubulin antibodies. Scale bar: 25 μm. **(E)** Expression of BAF45D and beta-actin was examined by IB assays using the indicated antibodies. **(F)** The total cell number in the NC siRNA group was defined as 100%. The cell numbers in the *Baf45d* siRNA group are shown as relative cell growth. **(G)** Mean cell growth in neuro2a cells. ^∗^*P* < 0.05, compared with that in the NC group. Statistical analysis involved the Mann–Whitney *U*-test and was carried out using SPSS 17.0 software.

To elucidate the role of BAF45D in neuronal cells, we transfected neuro2a cells, a neuroblastoma cell line, using control siRNA (NC) and *Baf45d* siRNA. Expression of BAF45D and beta-III-tubulin in the intact neuro2a cells was examined by IF assay (Figures 8A–D). Seventy 2 h after siRNA transfection, BAF45D protein is downregulated as compared to the NC control (Figure 8E). Moreover, cell growth of the neuro2a cells was examined at 1, 2, and 3 days after transfection. Compared to the NC siRNA, *Baf45d* siRNA decreased cell growth significantly (Figures 8F,G).

These results suggest that BAF45D knockdown inhibits cell growth in neuro2a cells.

## Discussion

In this study, we report the first expression profile of BAF45D in SCECs and spinal cord neurons in intact adult rats. Our findings concurred with our previous results in mice, which also demonstrated the expression of BAF45D in SCECs and spinal cord neurons of adult mice (Liu et al., 2017). Moreover, we found that astrocytes in the spinal cords of adult rats expressed little or no BAF45D. This is in accordance with data from the Human Protein Atlas showing that BAF45D is not detectable in the glial cells of the cerebral cortex^1^. We established a rat model of SCI in adult rats and found that SCI induced the proliferation of SCECs; this finding concurred with those of previous papers that also reported increases in the numbers of SCECs after SCI (Meletis et al., 2008; Sabelstrom et al., 2014; Cusimano et al., 2018). Surprisingly, despite the clear increase in the number of SCECs, the number of BAF45D-positive cells derived from the SCECs was significantly reduced. Given that SCI mainly induced SCECs to differentiate into glial cells (Becker et al., 2018), our data suggest that the cells derived from SCECs were predominantly glial cells. Because BAF45D is expressed in SCECs and neurons (Liu et al., 2017), this phenomenon suggests that BAF45D may also be involved in the proliferation of SCECs in response to SCI. Immunoblotting assays clearly demonstrated that the levels of BAF45D were downregulated at the lesion site. The loss of neurons at the lesion site was further confirmed by the downregulation of two neuronal markers: NEUN and beta-III-tubulin. We also detected the upregulation of GFAP, an astrocyte marker, in the new populations of cells. This finding concurs with the fact that SCI predominantly induces SCECs to differentiate into glial cells (Meletis et al., 2008). Previous research showed that SCI not only leads to the loss of neuronal cells, but can also disrupt neuronal connections and repair strategies, including the restoration of neuronal connections (O’Shea et al., 2017). Our data suggest that the beta-III-tubulin-positive CS-neurites may represent a potential *in vivo* index for monitoring the extent of neuronal connection after SCI. Given that the beta-III-tubulin has been used extensively by researchers to investigate axonal growth in cultured neurons (Gao and Chen, 2013; Zhou et al., 2014; Cawsey et al., 2015), it is possible that the CS-neurites may also be valuable for reflecting axonal growth in animals before and after SCI.

**FIGURE 9:**
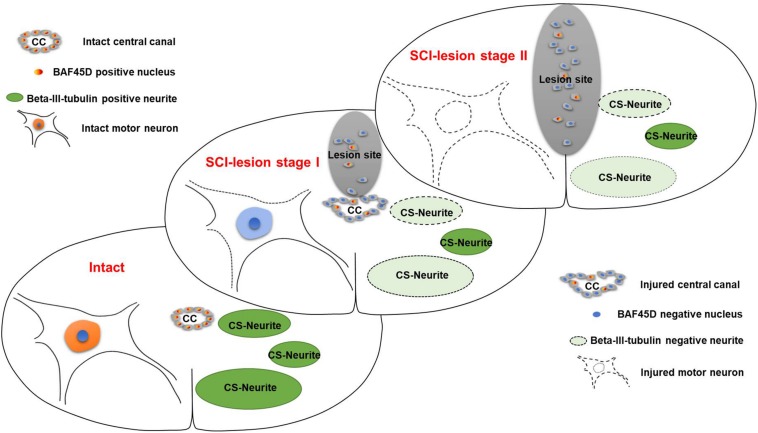
Schematic representation of the relationship between SCECs and neuronal lesions following SCI in adult rats. SCI-lesion stage I indicates the time point at which the CC became disorganized but the circular distribution of SCECs was retained. At this timepoint, the loss of motor neurons was moderate but there was a significant reduction in the number of neurites. SCI-lesion stage II indicates the time point at which there was a complete loss of SCEC organization together with a further loss of motor neurons, but not neurites, as compared with stage I. This suggests that SCECs protect SCI-lesions and that the loss of such protection accelerates the development of neuronal lesions. Moreover, the expression of BAF45D was downregulated in cells derived from SCECs, which proliferate and migrate to non-neural lesions during this process.

We also found that the disappearance of the CC was associated with a more significant reduction in the number of motor neurons in spinal cords, although the number of CS-neurites remained unchanged (Figure 9). Such changes were less evident when the CC was preserved. Consequently, the number of CS-neurites may represent a useful index for monitoring early lesions of the spinal cord *in vivo*. Previous research showed that the promotion of neurite outgrowth facilitates spinal cord regeneration *in vivo* (Cawsey et al., 2015). We therefore speculate that CS-neurites may also be useful when investigating the prognostic value of therapy for SCI. Furthermore, we also found, for the first time, that BAF45D is expressed in the myelin sheath of oligodendrocytes. The myelin sheath protects the axon and plays an important role in axonal regeneration after SCI (Assinck et al., 2017). The subcellular localization of BAF45D within the myelin sheath may help to elucidate the precise functional role of this protein in future studies.

It has been reported that SCECs become multipotent when isolated from SCI rats and can differentiate into reactive astrocytes (Takahashi et al., 2003) and oligodendrocytes (Moreno-Manzano et al., 2009). This is consistent with our present results in that SCECs were observed to undergo significant proliferation, and that the cells derived from this process were predominantly negative for both BAF45D and NEUN. Moreover, we found that the complete loss of the CC accelerated the loss of motor neurons. It is possible that the SCEC-derived scar component has several beneficial functions, including the restriction of tissue damage and neural loss following SCI (Stenudd et al., 2015). Indeed, NSCs are characterized by the formation of neurosphere when cultured *in vitro*; consequently, it is possible to demonstrate the presence of NSCs in adult SCECs using a neurosphere assay (Becker et al., 2018). Furthermore, both intact and SCI-injured SCECs can generate neurospheres capable of differentiating into functional spinal motor neurons *in vitro*, thus suggesting that the manipulation of endogenous SCECs could serve as a potential therapeutic strategy for the treatment of SCI (Moreno-Manzano et al., 2009).

Finally, we investigated the effect of BAF45D on the growth of neuro2a cells following the transfection of *Baf45d* siRNA. We found that the knockdown of BAF45D inhibited growth in neuro2a cells and may support the role of BAF45d in SCI-related neuropathology, at least in part. Previous research showed that *Baf45d* shRNA suppresses colony formation in two epithelial tumor cell lines, A549 and HeLa cells (Kobayashi et al., 2017). Furthermore, the stable expression of *Baf45d* shRNA expression in PANC-1 cells has also been shown to reduce colony formation (Tando et al., 2010). The similar role of BAF45D in the growth of both neuronal and non-neuronal cells indicates that BAF45D exerts a common function. However, the precise mechanisms underlying the functional role of BAF45D are yet to be elucidated. The neuro2a cell line exhibits the characteristic properties of neural progenitor/precursor cells in that cells can differentiate into neuron-like cells in the presence of retinoid acid (Nakamichi et al., 2012; Nakamura et al., 2012; Chaudhari and Ravanan, 2018; Sugahara et al., 2019). Consequently, the neuro2a cell line is commonly believed to represent a useful model for neural progenitor/precursor cells, and even adult neuronal precursors (Karl et al., 2005). In fact, loss of the neural progenitor/precursor pool is a key component of the neurogenic process and can be caused by the loss of two other BAF subunits, BAF170 and Ctip2 (Sokpor et al., 2017). Our data showed that the knockdown of BAF45D induced growth in neuro2a cells, thus suggesting that the loss of BAF45D may inhibit neuronal differentiation indirectly by reducing the pool of neural progenitor/precursor cells. However, whether BAF45D can directly manipulate the neuronal differentiation of SCECs remains an unanswered question and deserves further investigation. Our future research will therefore explore the direct effects of BAF45D on neuronal differentiation.

Recent research progress has shed significant light on the extrinsic and intrinsic factors that regulate the neurogenesis of multipotent ependymal cells and the implications of these ependymal cells in the repair of SCI (Qin et al., 2015). For example, in the CC of the macaque, it is known that ependymal cells with one or two cilia, but not multi-ciliated ependymal cells, can give rise to new ependymal cells. Moreover, it has been established that the infant and adult human spinal cord contains types of SCECs that resemble those present in the macaque (Alfaro-Cervello et al., 2014).

Collectively, our findings provide further understanding of the structural and biological roles of BAF45D in SCECs and SCI lesion in adult rats and identify a new target for SCI therapy via the manipulation of SCECs.

## Data Availability Statement

The datasets generated for this study are available on request to the corresponding author.

## Ethics Statement

The animal study was reviewed and approved by No. LLSC20190595 Laboratory Animal Ethics Committee of Anhui Medical University Project Demonstration Report (Original Copy) Project Director: Chao Liu. Project Title: Study of intrinsic factor BAF45D contributes to induction of neural stem cells mediate neuroregeneration after spinal cord injury through Smad3 signaling pathway. Project Organization: Anhui Medical University. (I) The committee have demonstrated the project and discussed three issues shown below. (1) Are the qualification of applicant, species or strain, grade and specifications of animals suitable? Could the quantity of animals be reduced by improving the study design or using high quality animals? (2) Does laboratory animal must be used in the project? Could other methods such as computer simulation, cell cultivation or using the low-grade animal instead of the high-grade animal? (3) Could the study design and animal treatment be refined by ameliorating experimental method, adjusting observational index, executing animal method? (II) The committee agree to implement the project according to the applicaton scheme. (III) Modifications and the contents and causes of the modification of the project should be demonstrated by the committee and put on records. (IV) Untoward effects of the projects during the implementation should be reported to the committee in writing. Sign: Qixing Zhu, committee director. Effective date: 2019.3.1 Laboratory Animal Ethics Committee of Anhui Medical University (official seal).

## Author Contributions

CoL conceived the study, designed the experiments, and wrote the manuscript. CoL, YS, and CS supervised the study. ZW and JH performed the experiments. CoL, ZW, and JH analyzed data. CgL contributed to supervision. LL contributed to animal experiment management.

## Conflict of Interest

The authors declare that the research was conducted in the absence of any commercial or financial relationships that could be construed as a potential conflict of interest.

## Footnotes

1 https://www.proteinatlas.org

## Abbreviations

BBB: Basso-Beattie-Bresnahan
BMP: bone morphogenetic protein
CC: central canal
DAPI, 4: 6-diamidino-2-phenylindole
IB: immunoblotting
IH: immunohistochemical
IF: immunofluorescence
MBP: myelin basic protein
SC: neural stem cell
PFA: paraformaldehyde
SCEC: spinal cord ependymal cell
SCI: spinal cord injury.
