# Supplementary material for: BAF45D Downregulation in Spinal Cord Ependymal Cells Following Spinal Cord Injury in Adult Rats and Its Potential Role in the Development of Neuronal Lesions
Source: Front Neurosci. 2019 Oct 29;13:1151. doi: 10.3389/fnins.2019.01151 (PMC6828649; doi:10.3389/fnins.2019.01151)
Supplement: Supplementary file 1 [file Table_1.DOCX]

**Supplement Table. 1 Description of the animals and sections for some of the experiments.**

| Experiments | Groups | Animals | Sections | Section location and areas |
| --- | --- | --- | --- | --- |
| Count of SCECs in CC* | Intact | #1 | 5 | Cross sections of intact T10, CC areas |
|  |  | #2 | 3 |  |
|  |  | #3 | 2 |  |
|  |  | #4 | 2 |  |
|  |  | #5 | 4 |  |
|  | SCI-10dpi | #1 | 3 | Cross sections of T10 lesion segments, CC areas |
|  |  | #2 | 2 |  |
|  |  | #3 | 2 |  |
|  |  | #4 | 2 |  |
|  |  | #5 | 3 |  |
|  |  | #6 | 3 |  |
| Count of neurites and neurons** | Intact | #1 | 4 | Cross sections of intact T10, anterior horns |
|  |  | #2 | 1^#^(2) |  |
|  |  | #3 | 5 |  |
|  |  | #4 | 3 |  |
|  | SCI-w-CC | #1 | 5 | Cross sections of T10 lesion segments, anterior horns |
|  |  | #2 | 3 |  |
|  |  | #3 | 5 |  |
|  |  | #4 | 3 |  |
|  | SCI-w/o-CC | #1 | 4 |  |
|  |  | #2 | 4 |  |
|  |  | #3 | 1^#^(2) |  |

* , for Figure 5I and J;**, for Figure 7 and Figure 8 A and B.

#, Two sections were employed. The anterior horns of one of the sections are not complete.
