# Supplementary material for: BAF45D Downregulation in Spinal Cord Ependymal Cells Following Spinal Cord Injury in Adult Rats and Its Potential Role in the Development of Neuronal Lesions
Source: Front Neurosci. 2019 Oct 29;13:1151. doi: 10.3389/fnins.2019.01151 (PMC6828649; doi:10.3389/fnins.2019.01151)
Supplement: Supplementary file 2 [file Table_2.DOCX]

**Supplement Table. 2 Description of the antibodies.**

| ***Name of the antibody*** | ***Catalog number*** | ***Host*** | ***Species specificity*** | ***Immunogen*** | ***Publications*** |
| --- | --- | --- | --- | --- | --- |
| BAF45D | 12111-1-AP | Rabbit | Human, mouse and rat | Ag2756* | ([1](#_ENREF_1)) |
| GFAP | 60190-1-Ig | Mouse | Human, mouse, rat and pig | Ag10452* | ([2](#_ENREF_2), [3](#_ENREF_3)) |
| NEUN | MAB377 | Mouse | Avian, chicken, ferret, human, mouse, pig, rat and salamander | Purified cell nuclei from mouse brain | ([4](#_ENREF_4), [5](#_ENREF_5)) |
| Beta-III-tubulin | MAB1637 | Mouse | Avian, pig, rat, bovine, sheep, human and monkey | Amino acids 443-450 of human beta-III-TUBULIN | ([6](#_ENREF_6), [7](#_ENREF_7)) |
| Beta-III-tubulin | ARG62683 | Mouse | Human, mouse, rat, pig, chick, bovine | Amino acids 441-448 of human beta-III-TUBULIN | ([8](#_ENREF_8)) |
| NESTIN | N5413 | Rabbit | Human, mouse, rat. | Amino acids 2554-270 of human NESTIN. | ([9](#_ENREF_9)) |
| GAPDH | 10494-1-AP | Rabbit | Human, mouse, rat and pig | Ag0766* | ([10](#_ENREF_10)) |
| MBP | AB62631 | Mouse | Mouse, Rat, Sheep, Rabbit, Goat, Human | Purified human myelin basic protein | ([11](#_ENREF_11)) |

* Cat number of Ptglab.

References:

1. Liu C, Zhang D, Shen Y, Tao X, Liu L, Zhong Y, et al. DPF2 regulates OCT4 protein level and nuclear distribution. *Biochim Biophys Acta* (2015) 1853(12):3279-93. Epub 2015/09/30. doi: 10.1016/j.bbamcr.2015.09.029

S0167-4889(15)00341-9 [pii]. PubMed PMID: 26417682.

2. Yin F, Guo L, Meng CY, Liu YJ, Lu RF, Li P, et al. Transplantation of mesenchymal stem cells exerts anti-apoptotic effects in adult rats after spinal cord ischemia-reperfusion injury. *Brain Res* (2014) 1561:1-10. Epub 2014/03/13. doi: 10.1016/j.brainres.2014.02.047

S0006-8993(14)00324-2 [pii]. PubMed PMID: 24613403.

3. Zhu Z, Liu Y, Li K, Liu J, Wang H, Sun B, et al. Protein tyrosine phosphatase receptor U (PTPRU) is required for glioma growth and motility. *Carcinogenesis* (2014) 35(8):1901-10. Epub 2014/05/31. doi: 10.1093/carcin/bgu123

bgu123 [pii]. PubMed PMID: 24876153.

4. Nair SG, Navarre BM, Cifani C, Pickens CL, Bossert JM, Shaham Y. Role of dorsal medial prefrontal cortex dopamine D1-family receptors in relapse to high-fat food seeking induced by the anxiogenic drug yohimbine. *Neuropsychopharmacology* (2011) 36(2):497-510. Epub 2010/10/22. doi: 10.1038/npp.2010.181

npp2010181 [pii]. PubMed PMID: 20962767; PubMed Central PMCID: PMC3005956.

5. Cai L, Bian M, Liu M, Sheng Z, Suo H, Wang Z, et al. Ethanol-induced neurodegeneration in NRSF/REST neuronal conditional knockout mice. *Neuroscience* (2011) 181:196-205. Epub 2011/03/15. doi: 10.1016/j.neuroscience.2011.02.059

S0306-4522(11)00229-6 [pii]. PubMed PMID: 21396985.

6. Scintu F, Reali C, Pillai R, Badiali M, Sanna MA, Argiolu F, et al. Differentiation of human bone marrow stem cells into cells with a neural phenotype: diverse effects of two specific treatments. *BMC Neurosci* (2006) 7:14. Epub 2006/02/18. doi: 1471-2202-7-14 [pii]

10.1186/1471-2202-7-14. PubMed PMID: 16483379; PubMed Central PMCID: PMC1397850.

7. Argenti B, Gallo R, Di Marcotullio L, Ferretti E, Napolitano M, Canterini S, et al. Hedgehog antagonist REN(KCTD11) regulates proliferation and apoptosis of developing granule cell progenitors. *J Neurosci* (2005) 25(36):8338-46. Epub 2005/09/09. doi: 25/36/8338 [pii]

10.1523/JNEUROSCI.2438-05.2005. PubMed PMID: 16148242.

8. Schmidt-Edelkraut U, Daniel G, Hoffmann A, Spengler D. Zac1 regulates cell cycle arrest in neuronal progenitors via Tcf4. *Mol Cell Biol* (2014) 34(6):1020-30. Epub 2014/01/08. doi: 10.1128/MCB.01195-13

MCB.01195-13 [pii]. PubMed PMID: 24396065; PubMed Central PMCID: PMC3958027.

9. Encinas JM, Enikolopov G. Identifying and quantitating neural stem and progenitor cells in the adult brain. *Methods Cell Biol* (2008) 85:243-72. Epub 2007/12/25. doi: S0091-679X(08)85011-X [pii]

10.1016/S0091-679X(08)85011-X. PubMed PMID: 18155466.

10. Wang P, Chen Z, Meng ZQ, Fan J, Luo JM, Liang W, et al. Dual role of Ski in pancreatic cancer cells: tumor-promoting versus metastasis-suppressive function. *Carcinogenesis* (2009) 30(9):1497-506. Epub 2009/06/24. doi: 10.1093/carcin/bgp154

bgp154 [pii]. PubMed PMID: 19546161.

11. Ganz J, Shor E, Guo S, Sheinin A, Arie I, Michaelevski I, et al. Implantation of 3D Constructs Embedded with Oral Mucosa-Derived Cells Induces Functional Recovery in Rats with Complete Spinal Cord Transection. *Front Neurosci* (2017) 11:589. Epub 2017/11/23. doi: 10.3389/fnins.2017.00589. PubMed PMID: 29163001; PubMed Central PMCID: PMC5671470.
