# Supplementary material for: BAF45D Downregulation in Spinal Cord Ependymal Cells Following Spinal Cord Injury in Adult Rats and Its Potential Role in the Development of Neuronal Lesions
Source: Front Neurosci. 2019 Oct 29;13:1151. doi: 10.3389/fnins.2019.01151 (PMC6828649; doi:10.3389/fnins.2019.01151)
Supplement: Supplementary file 3 [file Data_Sheet_1.PDF]

Supplementary Material

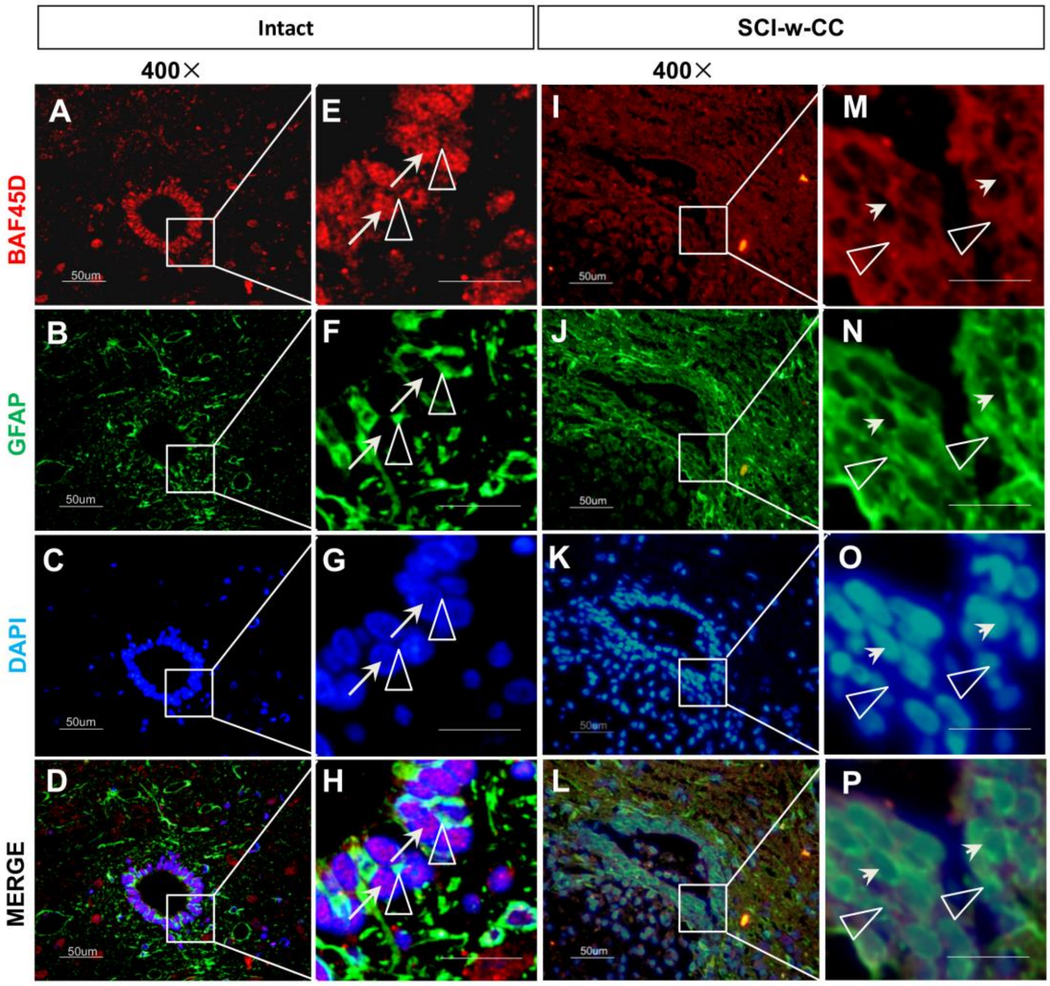

Figure S1. Expression of BAF45D and GFAP in the SCECs before and after the SCI.

A-H, BAF45D coexpresses with GFAP in the ependymal cells of the spinal cord CC of the adult rats. IF assay for the expression of BAF45D and GFAP in the SCECs of the intact CC in the adult rats (A-D). E, F, G and H are ~1900× magnifications of the insets in A, B, C and D, respectively. The triangles indicate expression of GFAP in cytoplasm. The arrows indicate presence of BAF45D in the nuclei. ***I-P,*** BAF45D is downregulated in the SCECs of the CC after SCI. IF assay for the expression of BAF45D and GFAP in the SCECS after SCI in the adult rats (I-L). M, N, O and P are ~1900× magnifications of the insets in I, J, K and L, respectively. The triangles indicate the expression of GFAP. The arrowheads indicate little or no expression of BAF45D in the nuclei. Bar=20μm (E-F and M-P).

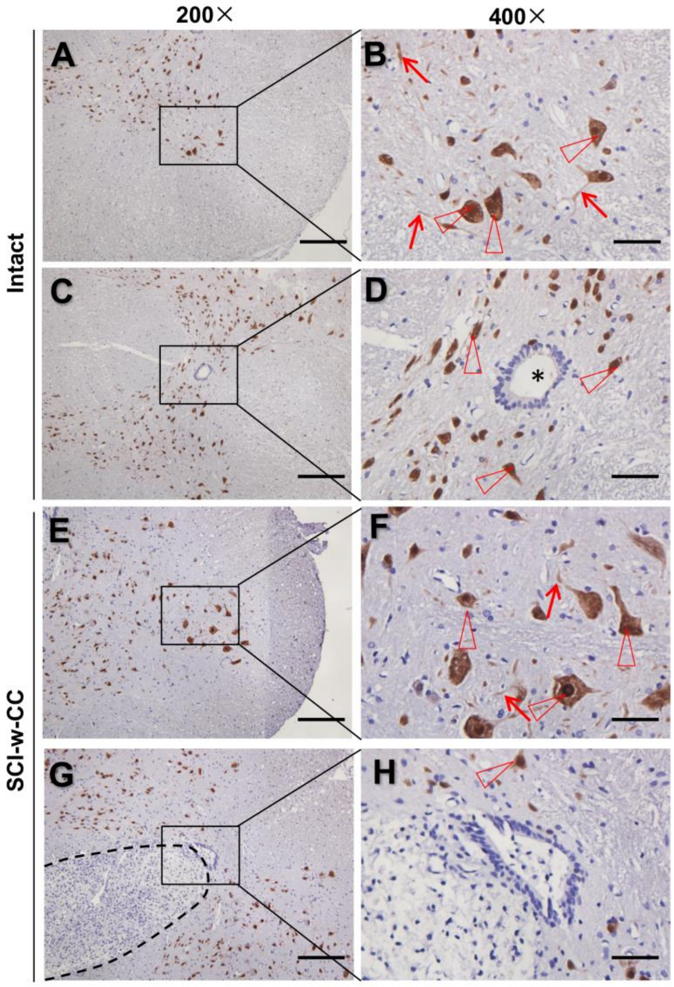

Figure S2. NEUN is expressed in a few of the neurites.

A-D, Expression of NEUN in the neurons in the intact spinal cords. B and D are higher magnifications of the inlets in A and C, respectively. ***E-H,*** Expression of NEUN in the neurons in the injured spinal cords. F and H are higher magnifications of the inlets in E and G, respectively. The region with a dashed line indicates the lesion site (G). Bar=100µm (A, C, E and G). Bar=25µm (B, D, F and H). The magnifications are also shown.

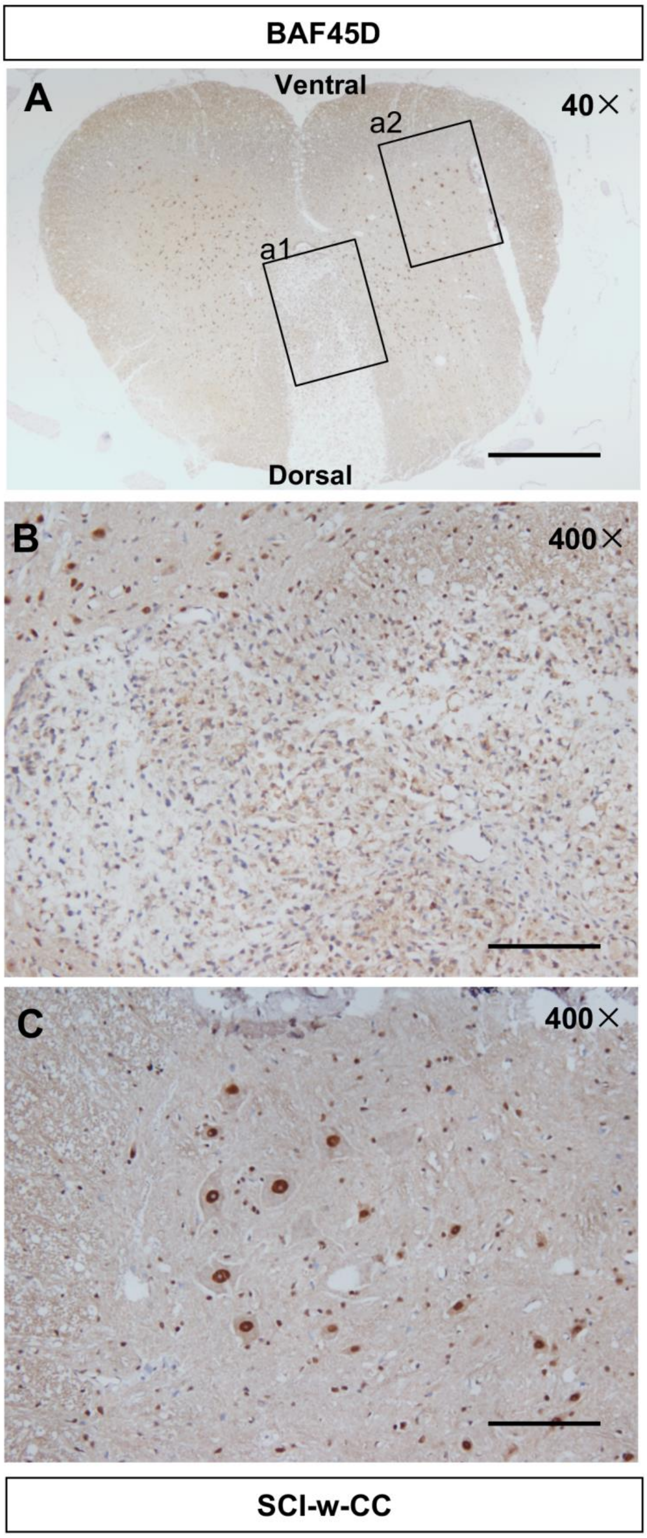

Figure S3. Weak expression of BAF45D was detected in the non-neuronal lesion region after SCI in the adult rat spinal cords.

A, The cross section of the injured spinal cord was subjected to IH assay using anti-BAF45D antibodies. ***B*** is a 400× magnification of the inset a1 in A. ***C*** is a 400× magnification of the inset a2 in A. Bar=500 μm (A) and 50 μm (B and C). The magnifications are also shown.

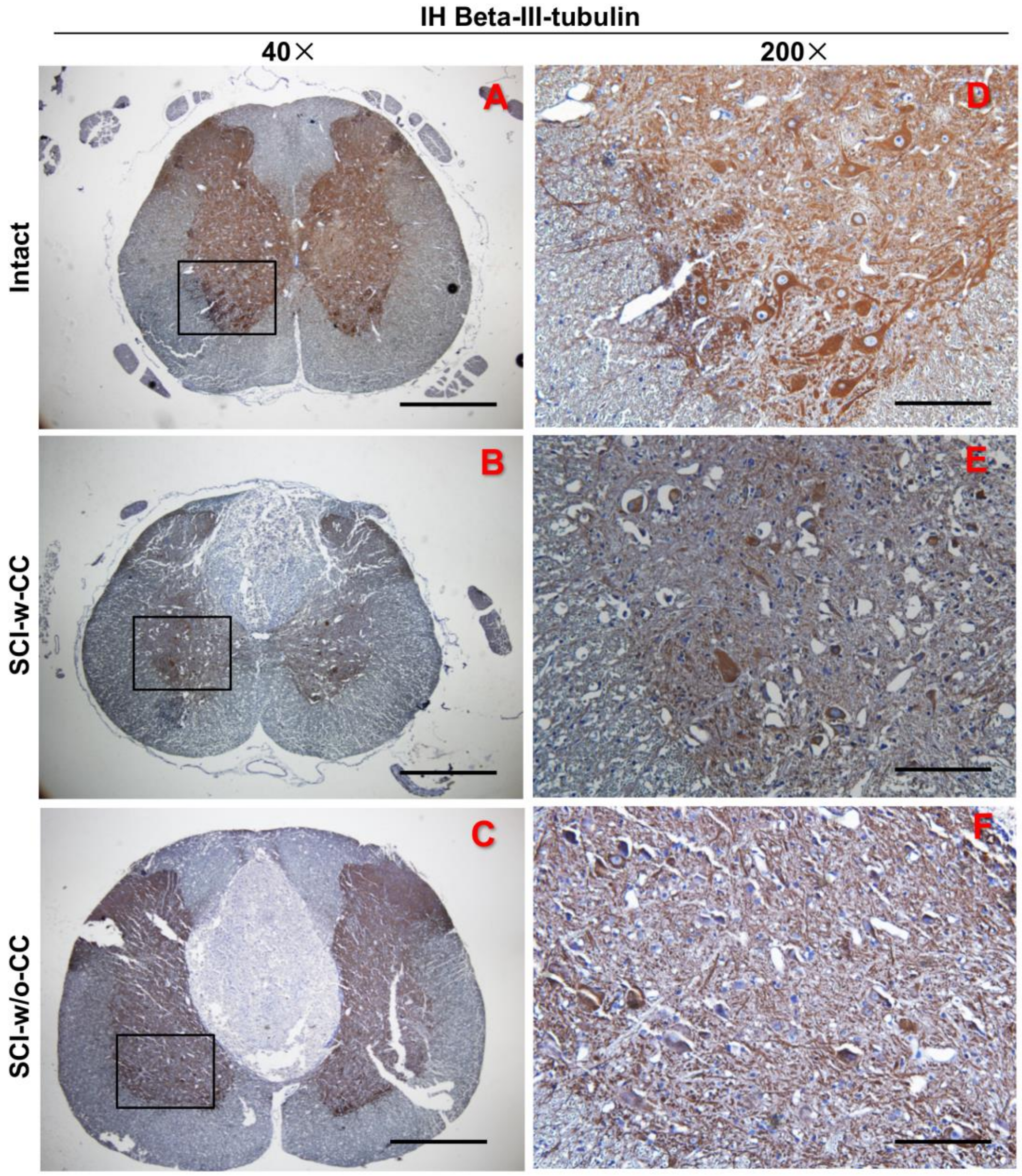

Figure S4. Expression of beta-III-tubulin in the complete cross sections of intact and SCI-injured spinal cords.

A-C, Immunohistochemistry assay using anti-beta-III-tubulin antibodies in the complete cross sections of the spinal cords from intact, SCI-w-CC and SCI-w/o-CC groups. D, E and F are 200× magnifications of the anterior horn areas in A, B and C, respectively. Bar=500 μm (A-C), 100 μm (D-F).The magnifications are also shown.

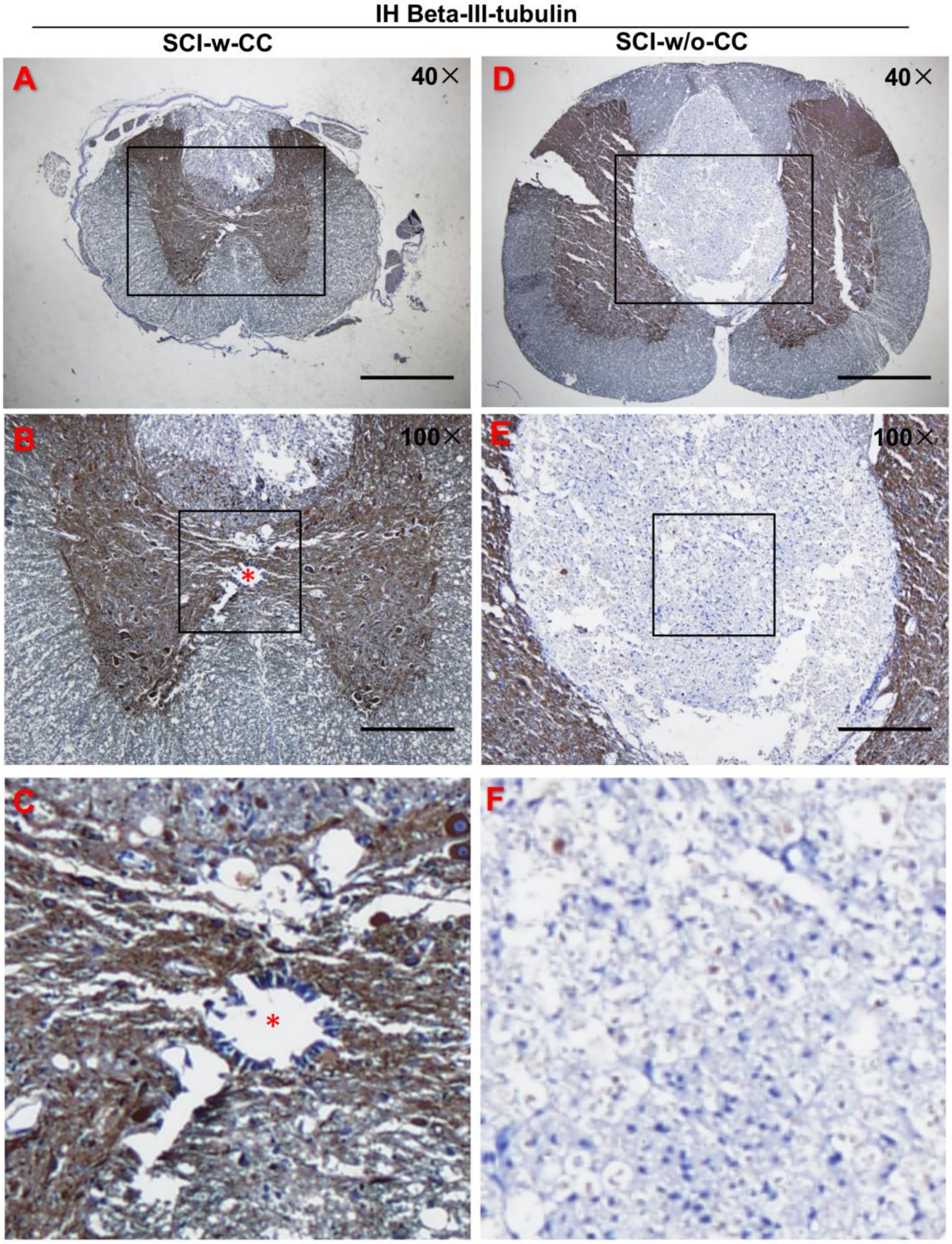

Figure S5. Loss of CC structure in the SCI-injured spinal cords.
A-C, Immunohistochemistry assay using anti-beta-III-tubulin antibody in cross sections of the spinal cords from SCI-w-CC group. B is 100× magnification of the inset in A. C is 400× magnification of the insets in B. The asterisk indicates the presence of CC despite its structure is not complete. ***D-F***, Immunohistochemistry assay using anti-beta-III-tubulin antibody in cross sections of the spinal cords from SCI-w/o-CC group. E is 100× magnification of the inset in A. C is 400× magnification of the inset in B. No CC structure can be identified. Bar=500 μm (A and D), 200 μm (B and E).The magnifications are also shown.
